# Imaging Modalities for the Diagnosis of Vascular Graft Infections: A Consensus Paper amongst Different Specialists

**DOI:** 10.3390/jcm9051510

**Published:** 2020-05-17

**Authors:** Chiara Lauri, Roberto Iezzi, Michele Rossi, Giovanni Tinelli, Simona Sica, Alberto Signore, Alessandro Posa, Alessandro Tanzilli, Chiara Panzera, Maurizio Taurino, Paola Anna Erba, Yamume Tshomba

**Affiliations:** 1 Nuclear Medicine Unit, Department of Medical-Surgical Sciences and of Translational Medicine, “Sapienza” University of Rome, 00161 Rome, Italy; alberto.signore@uniroma1.it; 2 Department of Nuclear Medicine and Molecular Imaging, University of Groningen, University Medical Center Groningen, 9700 Groningen, The Netherlands; paola.erba@unipi.it; 3 Radiology Unit, Fondazione Policlinico Universitario A. Gemelli IRCCS, Roma-Università Cattolica del Sacro Cuore, 00168 Rome, Italy; roberto.iezzi@unicatt.it (R.I.); alessandro.posa@gmail.com (A.P.); alessandrotanzilli93@gmail.com (A.T.); 4 Radiology Unit, Department of Medical-Surgical Sciences and Translational Medicine, Faculty of Medicine and Psychology, Sant’Andrea Hospital, “Sapienza” University of Rome, 00161 Rome, Italy; michele.rossi@uniroma1.it; 5 Unit of Vascular Surgery, Fondazione Policlinico Universitario Gemelli IRCCS, Roma-Università Cattolica del Sacro Cuore, 00168 Rome, Italy; giovanni.tinelli@policlinicogemelli.it (G.T.); simonasica1@gmail.com (S.S.); yamume.tshomba@policlinicogemelli.it (Y.T.); 6 Vascular Surgery Unit, Department of Clinical and Molecular Medicine, Faculty of Medicine and Psychology, Sant’Andrea Hospital, “Sapienza” University of Rome, 00161 Rome, Italy; chiara.panzera1@gmail.com (C.P.); maurizio.taurino@uniroma1.it (M.T.); 7 Nuclear Medicine, Department of Translational Research and New Technology in Medicine, University of Pisa, 56123 Pisa, Italy

**Keywords:** infection, vascular graft, multimodality imaging, WBC scintigraphy, FDG-PET/CT, angio-CT, personalized medicine

## Abstract

Vascular graft infection (VGI) is a rare but severe complication of vascular surgery that is associated with a bad prognosis and high mortality rate. An accurate and prompt identification of the infection and its extent is crucial for the correct management of the patient. However, standardized diagnostic algorithms and a univocal consensus on the best strategy to reach a diagnosis still do not exist. This review aims to summarize different radiological and Nuclear Medicine (NM) modalities commonly adopted for the imaging of VGI. Moreover, we attempt to provide evidence-based answers to several practical questions raised by clinicians and surgeons when they approach imaging in order to plan the most appropriate radiological or NM examination for their patients.

## 1. Introduction

Vascular graft infection (VGI) is a rare condition, representing one of the most life-threatening complications in vascular surgery. The incidence ranges from 1.5% to 6%, mainly depending on the anatomic location of the graft, and clinical characteristics are highly variable and are related to the site of the implant, causative pathogen, and time after surgery [1].

Location categories for VGI include extracavitary (primarily in the groin—80%, or lower extremities—20%) and intracavitary (primarily in the abdomen—70%, or less commonly within the thorax—30%) sites. Extracavitary infections usually occur when there is a wound infection in the groin or intraoperative contamination, while intracavitary infections are due to intraoperative contamination, mechanical erosion in the bowel, genitourinary system or skin seeding by bacteremia, or involvement in contiguous infectious processes such as spondylodiscitis.

According to the time of onset after surgery, VGIs may be classified in “early” infections if they occur within 4 months after implantation and they usually show systemic signs and symptoms of infection (as fever); or “late” infections when they occur after 4 months from surgery and, in this case, signs and symptoms could be absent [2,3].

Patient related risk factors are diabetes, malnutrition, chronic renal impairment/failure, liver disease/failure or cirrhosis, previous radiotherapy or chemotherapy, malignancy, autoimmune disorders, long term corticosteroid use [4].

Diagnosis of VGI is complex, being related to clinical presentation, laboratory studies and imaging, so quick and correct diagnosis of VGIs can be challenging.

Standard laboratory tests are usually non-specific: typical findings include leukocytosis (left shift) and a high erythrocyte sedimentation rate. Cultures from wounds or perigraft fluid can be collected in VGI-suspected patients in order to diagnose and guide antibiotic therapy [5].

The Management of Aortic Graft Infection Collaboration (MAGIC) depicted major and minor criteria for VGI diagnosis, based on clinical/surgical, laboratory and radiological data: aortic graft infection (AGI) can be suspected when there is one major criterion, or two minor criteria from two different categories, whereas diagnosis is certain if there is one major criterion plus any other criterion (both minor or major) from another category [6].

A prompt identification of the infection and its extent is crucial for prognostication of the patient and for planning the correct treatment. Although there is general agreement that the diagnosis of VGI derives from a combination of clinical, radiological, nuclear medicine (NM) and laboratory findings, an univocal consensus on the diagnostic criteria for imaging modalities still does not exist.

This review aims to provide an updated overview of radiologic and NM strategies for the diagnosis of VGI.

## 2. Surgical Management of VGI: How Can Imaging Be Helpful?

**Figure 1:**
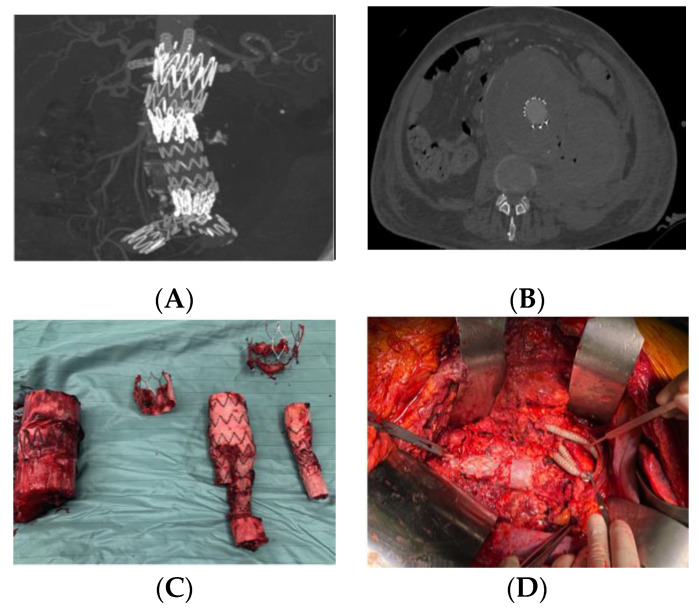
(**A**,**B**) Pre-operative computed tomography (CT) scan showing graft disruption, perigraft fluid and air in a 72-year-old man with an infected abdominal endograft; (**C**) explanted graft after in situ repair (ISR) with visceral debranching: aorto-mesenteric bypass, right renal artery Y graft, aorto-left renal artery bypass. The reconstruction has been completed with lower extremity revascularization with extra-anatomic reconstruction (EAR) axillo-bifemoral; (**D**) final result after EAR.

The management of VGI is extremely complex, and the centralization of the patient is crucial. The treatment needs to be evaluated on a case-by-case basis. Antimicrobial therapy is an integral part of VGI treatment. In the acute phase, intensive antimicrobial therapy with (broad range) antibiotics, directed against the most likely infecting organisms, is indicated to control infection and sepsis [7]. However, when possible, surgical therapy must be attempted. Recently, the European Society for Vascular Surgery (ESVS) 2020 Clinical Practice Guidelines on the Management of Vascular Graft and Endograft Infections recommended the complete excision of all graft material and infected tissue for fit patients (Class I, Level B) [7]. Historically, the gold standard surgical approach was the total removal of the infected graft, extensive debridement of the infected area, and extra-anatomic reconstruction (EAR) outside the infected field. However, this approach has higher 30-day mortality (26.7%) and lowest one-year survival (54.3%) rates compared to in situ repair (ISR) [8]. Indeed, nowadays, most surgeons prefer the second approach. Like the original gold standard, ISR includes complete removal of the graft, aggressive debridement of the infected tissues, but, unlike EAR, ISR also provides arterial reconstruction with suturing in the healthy, non-infected aorta (Figure 1). A video about graft removal is available in supplementay materials (Video S1).

Different graft materials may be used for reconstruction, including autologous veins, cryopreserved allografts, rifampicin-bonded or silver-coated synthetic grafts, and xenogenous grafts, and they seem to show similar rates of infection (veins 2%, cryopreserved allografts 9%, rifampicin bonded or silver coated prosthesis 11%) [9].

Imaging plays a key role in confirming the diagnosis of VGI and guiding the treatment. In particular, it is useful in investigating the position and the structural integrity of the graft or endograft (Figure 1), it may confirm or exclude peri-graft inflammation, and delineate its extent; it may reveal the presence or absence of perigraft fluid or gas, anastomotic leakage or pseudoaneurysms, the grade of graft involvement and the presence of graft-enteric erosion/a fistula. Moreover, imaging is fundamental to plan a strategy for revascularization, and for imaging-guided perigraft fluid aspiration.

## 3. Radiological Modalities for Imaging VGI

### 3.1. Ultrasonography (US)

Ultrasonography (US) is the first-choice imaging modality for assessing vascular diseases due to its well-known advantages, represented by repeatability, availability, cost-effectiveness, safety profile; moreover, it is non-invasive and easy to perform. However, it is operator dependent, and is hampered by patient habitus (obesity, intestinal gas or ascites) and the patient’s level of collaboration. In addition, it doesn’t offer a detailed anatomic roadmap like other imaging modalities. US can be useful in the evaluation of perigraft fluid collections or abscesses, and can distinguish a fluid collection from a hematoma or a pseudoaneurysm. It can also be used for US-guided aspiration [10]. Contrast-Enhanced Ultrasonography (CEUS) is not routinely used for the diagnosis of VGI, due to its unproven ability to improve diagnostic performance [11].

### 3.2. Computed Tomography (CT)—CT–Angiography (CTA)

Computed tomography (CT) is the first-choice and gold-standard imaging modality, particularly in intracavitary VGI. A recent meta-analysis showed that CT–angiography (CTA) has an overall pooled sensitivity of 67% and an overall pooled specificity of 63% [12]. In particular, previous studies depicted a difference in the diagnostic performance of CTA between low- and high-grade VGI.

**Figure 2:**
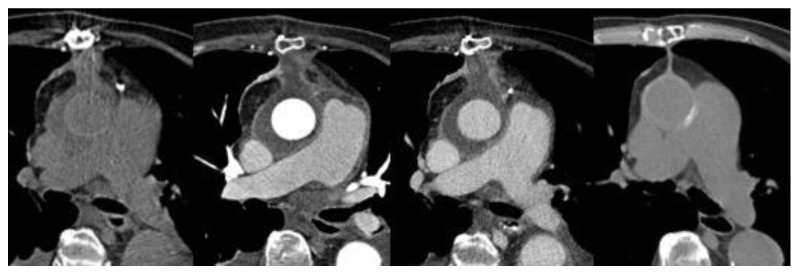
Post-surgical ascending aortic repair (Bentall procedure) 1-month CT scan. From left panel to right: unenhanced CT, arterial phase and late phase CT images show aortic graft patency with perigraft fluid and stranding. These findings can be considered a typical post-operative appearance as confirmed by their disappearance in the 3-month unenhanced CT image (right image).

CT in low-grade infections has high false negative rates, resulting in a sensitivity of only 55.5% [6], since it can be very difficult to differentiate early/low-grade VGI findings from para-physiological ones (e.g., the postoperative local residues as small fluid collection or gas). It is not clearly defined at what time after surgery the presence of gas or fluid can be considered to represent suspected/positive VGI (Figure 2). On the other hand, CT has better accuracy in advanced or complex VGI (e.g., aorto-enteric erosion/fistula), with a sensitivity and specificity of about 85–94% [6,12].

However, radiological follow-up could be mandatory for increasing diagnostic accuracy in VGI. In detail, on serial CTA follow-up, a suspect can be posed if there are new findings, or the perigraft fluid/gas collection increases over time or persists beyond three months from the surgery, or there is a rapid dimensional increase in the aneurysm sac [13,14,15].

**Figure 3:**
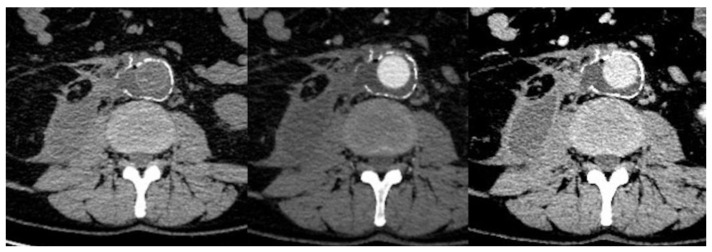
Open surgical repair of abdominal aortic aneurysm (65-year-old male). From left panel to right: unenhanced and enhanced (arterial phase and late phase) CT scans 4 months after treatment show aortic graft patency with perigraft fluid and air, enhancing the soft tissue around the graft and abscess near the right psoas muscle. These findings are consistent with perigraft infection, as also confirmed by fluid aspiration.

MAGIC minor criteria alone are not sufficient for the diagnosis of VGI, due to their subjective nature; these minor criteria include perigraft soft tissue alterations, like fat stranding (pathological increase in fat tissue attenuation) and phlegmon (diffuse inflammation of the soft or connective tissue) [16,17]. Infection spreading to adjacent structures can cause hydronephrosis, psoas abscess, focal bowel thickening, and discitis/osteomyelitis, but the presence of a major criterion is required to confirm the VGI [18,19] (Figure 3).

Pseudoaneurysm formation is a recognized finding in VGI, but it may also be present in a non-infective setting, particularly following the focal dehiscence of a vascular suture. Septic emboli from the infected graft can be a threatening occurrence, leading to vascular occlusions and the distal spread of the infection [20].

**Figure 4:**
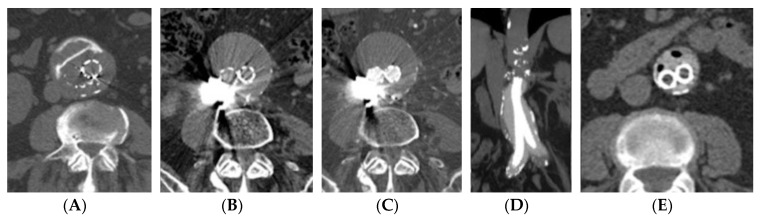
In patients with aortic stent grafts who underwent embolization for type II endoleak, diagnostic pitfalls need to be considered and known. They could be represented by hyperdense structures/materials, represented by glue/liquid embolics or coils (**A–C**), or also by intra-sac gas, in the case of percutaneous puncture/embolization, or new endografts with polymer-filled endobags (**D–E**).

VGI must not be confused with the primary vasculitis of large vessels, even though these two entities are unlikely to be similar, the latter not usually being localized around the graft and being associated with wall-thickening [21]. Chapman et al. reported the case of a VGI mimicking hypertrophic osteoarthropathy [22]. Imaging alone, however, can be deceitful, due to the presence of some diagnostic pitfall conditions (mostly iatrogenic) that can mimic VGI. In more detail, in patients who underwent periaortic fluid aspiration or in patients with an aortic endograft and a recent type-II endoleak embolization with the direct puncture of the aneurysmatic sac, gas-like images could represent diagnostic pitfalls, mimicking graft infections. For these reasons, an adequate clinical history knowledge, including all procedures performed, is mandatory to avoid false-positive diagnosis. Performing nonenhanced imaging is particularly important in this postoperative setting, as surgical or embolic devices (glue, coils, or other high-attenuation materials) may be most conspicuous at this phase. Furthermore, some bioabsorbable hemostatic agents such as gelatin or cellulose may also appear as an ill-defined, gas-filled heterogeneous mass, sometimes with rim enhancement, potentially mimicking abscesses, hematomas, or retained foreign bodies (Figure 4). In selected cases, CT can be also used to guide the percutaneous aspiration of perigraft fluid collections.

### 3.3. Magnetic Resonance Imaging (MRI)

Magnetic resonance imaging (MRI) has not been evaluated as extensively as CT for the diagnosis of VGI, but has demonstrated good positive and negative predictive values (95% and 80% respectively) [11]. MRI has some advantages compared to CT examination, due to the absence of radiation exposure, the use of noniodinated contrast media, and the possible application of advanced imaging techniques (e.g., functional and dynamic imaging). However, it has some disadvantages like a longer examination time, less availability, and higher costs. Moreover, a high magnetic field strength is required with an increase in ferromagnetic artifacts due to metallic stents. When considering safety issues, it is well known that risk of incompatibility is quite low, as MR-compatible materials have been increasingly used since the mid-1990s. Most vascular grafts are mainly made of stainless steel or nitinol, are non-ferromagnetic, or contain variable amounts of platinum, cobalt alloy, gold, tantalum, making them weakly ferromagnetic. Furthermore, implantation against the vessel wall provides sufficient stability, reducing the risk of dislodgement. Data from the literature allow us to conclude that MRI can be performed in patients after vascular graft implantation without significant risk at any time, but the risk of incompatibility must be well known and properly checked. Claustrophobia, pacemakers or patients not compliant with sedation (<5%) are contraindications to MRI. MRI, with its high contrast resolution, can easily demonstrate small perigraft fluid collections but, like CT, it is not able to distinguish the para-physiological perigraft fluid in the early postoperative period from an infected perigraft fluid collection. MRI imaging does not allow for the differentiation of the signal void produced by calcifications of the aortic wall from that of air bubbles in the perigraft infection [23]. In the case of graft infection, MRI can show eccentric fluid collection with low to medium signal intensity on T1-weighted images and high signal intensity on T2-weighted ones.

MRI is able to better distinguish perigraft fluid from inflammation and fibrosis than CT [18]. The use of contrast-enhanced T1-weighted fat acquisitions may also help in detecting the surroundings of tissue edema and inflammatory alteration that are indicative but non specific findings of infection.

### 3.4. Digital Subtraction Angiography (DSA)

DSA has a role for revascularization in selected patients (e.g., in case of distal limb or splanchnic ischemia, occlusive disease or graft thrombosis), and to better define inflow and outflow targets for the surgical bypass. It is mandatory for interventional procedures, whereas it has almost no use in VGI diagnosis.

## 4. Nuclear Medicine Imaging of VGI

Functional hybrid imaging offers the possibility to study a process from a molecular point of view and it is able to identify pathophysiological signs that can occur before morphological changes become detectable.

Different radiopharmaceuticals and modalities are available for imaging infection and inflammation. In particular, in suspected VGI, two procedures are currently applied, radiolabeled white blood cells (WBC) scintigraphy and ^18^F-fluorodeoxyglucose positron emission tomography/computed tomography ([^18^F]FDG PET/CT).

### 4.1. Gamma-Camera Imaging for VGI

The role of radiolabeled WBC scintigraphy in the field of infection is nowadays well consolidated. The possibility to specifically investigate granulocyte migration in tissues represents a surrogate marker of infections [24]. It provides an accurate differentiation between infection and sterile inflammation. This imaging modality is, therefore, considered the gold standard for the diagnosis of several infective diseases [25].

Granulocytes can be easily radiolabeled with both ^111^In and ^99m^Tc, with the latter being the preferred isotope for both physical characteristics and dosimetric issues.

**Figure 5:**
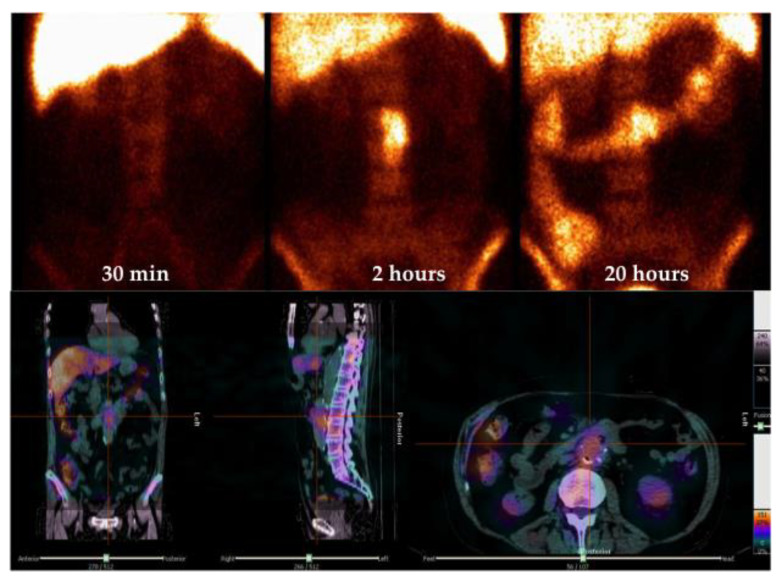
An example of ^99m^Tc-labeled white blood cell (WBC) scintigraphy acquired with times corrected for isotope decay at 30 min, 2 and 20 h p.i. in a patient with suspected abdominal vascular graft infection (VGI). Planar anterior images show an increased uptake in abdominal region that was consistent for an infection (upper panel). Dingle-photon emission computed tomography (SPECT)/CT images (bottom) acquired 2 h p.i. allowed the correct localization of the uptake in the inner of abdominal aortic graft.

The European Society of Nuclear Medicine (EANM) provided several guidelines to address the standardization of WBC labeling, acquisition protocols and interpretation criteria [25,26,27]. In particular, for the assessment of VGI, a dynamic scan within the first 5 min is suggested in order to visualize the vascular tree and aneurisms. Static images acquired, with times corrected for the isotope decay, at 30 min–1 h (early images) post injection (p.i.) and delayed images (2–4 h p.i.), might be sufficient to provide the diagnosis. However, late images (20–24 h p.i.) are strongly recommended in equivocal cases, low grade/chronic infection and follow-up studies [25,28] when positive single-photon emission computed tomography (SPECT)/CT images are mandatory for the exact localization of the infection (soft tissue only, graft, or both) and for the evaluation of its extent (7) (Figure 5), since their use has been demonstrated as increasing the diagnostic accuracy [25,28].

A whole-body scan at 2–4 h p.i. is strongly suggested in order to detect any additional sites of infection or septic embolism.

Once correctly acquired and displayed, the correct interpretation is derived from the comparison of uptake extent and intensity between late and delayed images. By following these recommendations, we can easily differentiate between an infection from sterile inflammation, which is an infection characterized by an increased uptake over time in terms of extent and/or intensity, and sterile inflammation characterized by a decreased or stable uptake over time [25,29,30].

Data from the literature are inconsistent, with different accuracies being reported, depending on the population sample and the different method and gold standard used. However, if we take into account the few existing meta-analyses and systematic reviews on this imaging modality, the authors all conclude that radiolabeled WBC is a powerful tool in diagnosing a VGI [12,31] (Table 1).

In particular, comparing ^99m^Tc-WBC scintigraphy with ^111^In-WBC scintigraphy and CT, Annovazzi et al. [31] reported higher sensitivity (97.7% vs. 84.1% vs. 75%), specificity (88.6% vs. 79.4% vs. 56.6%), diagnostic accuracy (94.6% vs. 81.5% vs. 78.6), positive predictive value (PPV) (90% vs. 85% vs. 100%) and negative predictive value (NPV) (100% vs. 93.8% vs. 82%) for the ^99m^Tc-WBC scan. More recently, in another meta-analysis, WBC SPECT/CT demonstrated the highest diagnostic performance in VGI diagnosis [12] (Table 1). Indeed, the added value of SPECT/CT over planar images has been clearly shown by several authors. In the study performed by Bar-Shalom and co-workers, ^111^In-WBC SPECT/CT was able to improve diagnosis, better localize and evaluate the extent of the disease in 67% of patients with suspected VGI [32]. These results were further confirmed in the retrospective study performed by Khaja and colleagues on 20 patients with suspected VGI where the use of SPECT/CT resulted in improved sensitivity, diagnostic accuracy and NPV compared to the planar images and standalone CT [33].

Similarly, in 55 patients with suspected late and low-grade VGI, ^99m^Tc-WBC SPECT/CT showed a specificity and sensitivity of 100%, far superior to planar images, SPECT stand alone and ultrasounds (US), reducing false positive results in 37% of patients [28]. The estimated sensitivity of WBCS (without SPECT/CT) in diagnosing VGEI in the most recent meta-analysis was 0.90 (95% CI 0.85 to 0.94) with a specificity of 0.88 (95% CI 0.81 to 0.94) [12]. When WBCS was combined with SPECT/CT, the sensitivity increased to 0.99 (95% CI 0.92 to 1.00), with a specificity of 0.82 (95% CI 0.57 to 0.96) (Table 1).

Several factors, unfortunately, limit the routine use of radiolabeled WBC in clinical practice—the labeling procedure is time consuming and it requires the manipulation of potentially infected blood. For these reasons, the labeling procedure must be performed by trained personnel in dedicated environments (with isolators, laboratories equipped with hoods and centrifuges). Moreover, multiple timepoints are necessary for the acquisitions, thus requiring the patient to come back to the NM Department the day after in order to conclude the exam. Because of the aforementioned limitations and limited availability, the recent European Society for Vascular Surgery 2020 Clinical Practice Guidelines on the Management of Vascular Graft and Endograft Infections does not recommended WBCs as the first imaging modality in diagnosing VGI [7].

Scintigraphy with radiolabeled anti-granulocyte antibodies (AGA) has been investigated in alternative WBC scintigraphy.

In VGI, some series reported a sensitivity of 92–100% and a specificity ranging from 62.5% to 100% [34,35,36,37]. The in vivo labeling procedure of a murine AGA is easier and quicker compared to the in vitro labeling of WBC. However, the main drawback of this approach is related to the possibility to induce human anti-murine antibodies (HAMA) after the administration of these molecules, thus limiting their use in follow-up. Moreover, the data available in the literature on the use of radiolabeled AGA in the assessment of VGI are based only on small series without standardized protocols of acquisition and interpretation. Therefore, there is no convincing evidence supporting their superiority over autologous leucocytes.

### 4.2. [^18^F]FDG PET/CT Imaging of VGI

In the last decades, [^18^F]FDG PET/CT has gained an important role in the field of infection and inflammation, as summarized in the guidelines published in 2013 by EANM and the Society of Nuclear Medicine and Molecular Imaging (SNMMI) [40].

[^18^F]FDG PET/CT offers several advantages over labeled WBC: the presence of a CT co-registration that does not require any change in the patient’s position and which allows a more precise localization of the uptake and higher quality images than gamma camera isotopes. Moreover, the length of scan is shorter (2–3 h vs. 20 h) and it provides a whole-body study without the need for blood manipulation.

Despite a high sensitivity, a major drawback of [^18^F]FDG is its relatively low specificity. False positive results may be observed in post-surgical flogosis, especially within the first 6−8 weeks, and in foreign-body reaction induced by the synthetic materials of the graft, characterized by a low-grade inflammation [41,42]. Therefore, to limit the rate of false positive results, specific interpretation criteria need to be applied.

Table 1 summarizes the results of the most recent meta-analyses evaluating [^18^F]FDG PET/CT in the work-up of VGI [12,38,39]. The most recent one [38] reports a pooled sensitivity of 96%, ranging between 81% [43] and 100% [42,44,45,46], and a pooled specificity of 74%, ranging between 29% [47] and 92% [48].

Several interpretation criteria for [^18^F]FDG PET/CT have been proposed and they mainly consider the pattern of uptake, the tissue to background (T/B) ratio, the visual grading scale and the calculated maximum standardized uptake value (SUVmax). The [^18^F]FDG pattern can be classified as “focal” or “diffuse”, “homogeneous” or “inhomogeneous”, “mild” or “intense”.

Spacek et al., studying 96 low-grade prostheses, defined focal uptake as the most valid diagnostic parameter, leading to a very high specificity (92.7%) and PPV (93.5%). Conversely, mild inhomogeneous uptake must be interpreted with caution, being consistent with both low-grade infection and sterile inflammation around the foreign body. The co-registered CT assessment for the definition of graft borders (irregular vs. smooth) is also of paramount importance in this manuscript: the presence of irregular borders associated to focal [^18^F] FDG uptake is highly predictive of VGI [41].

**Figure 6:**
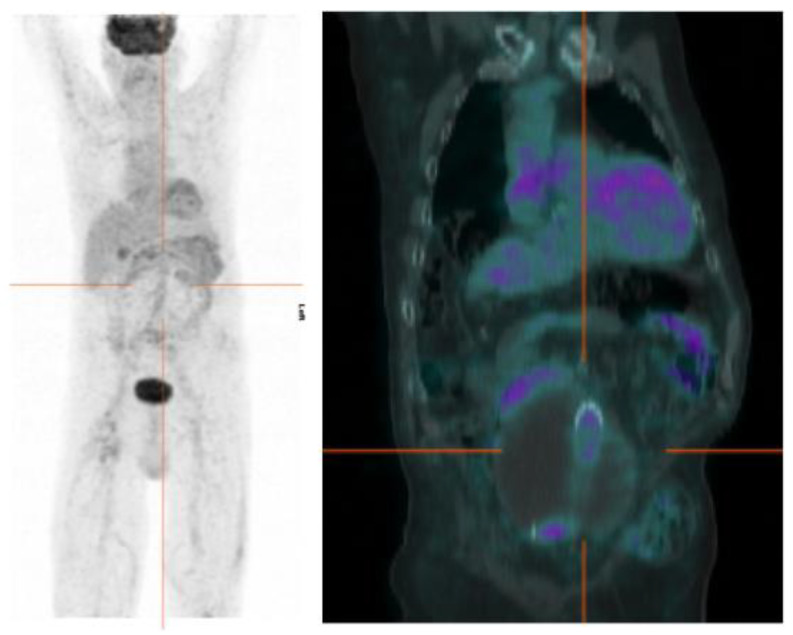
An example of negative [^18^F]FDG PET/CT scan in a patient with suspected infection of abdominal graft implanted 2 years before for the exclusion of a large aneurysm. The images show mild (SUVmax 2.4), homogeneous uptake along the whole tract of the prosthesis without any focal uptake.

Focal uptake as major sign of VGI, compared to the diffuse homogeneous uptake found in up to 92% of non-infected grafts and most frequently observed in Dacron prostheses (Figure 6), has also been reported by Keidar et al. [49].

A four- or five-point visual scale was also proposed to diagnose VGI, with the presence of grades 3 or 4 being indicative of infection [50,51]. In grade 0, [^18^F]FDG uptake is similar to background uptake; in grade 1, [^18^F]FDG uptake is similar to that which occurs in the inactive muscles and fat (low [^18^F]FDG uptake); in grade 2, [^18^F]FDG uptake is higher than in inactive muscles (moderate [^18^F]FDG uptake); in grade 3, [^18^F]FDG uptake is less than the physiologic uptake shown by the bladder (strong [^18^F]FDG uptake) and in grade 4, [^18^F]FDG uptake is comparable to physiologic urinary uptake (very strong [^18^F]FDG uptake).

The contributions of SUVmax and T/B ratio in the qualitative evaluation have also been examined.

Several thresholds of SUVmax have been proposed, but they are not universally recognized. Some authors suggested a SUVmax > 8 in the perigraft area as cut-off value for distinguishing infected graft from non-infected graft [42,50]. The use of this cut-off was associated with 100% of specificity and 80% of sensitivity. However, since it is well known that SUVmax evaluation is affected by several technical factors that may differ among the centers, T/B ratio is maybe a more reproducible parameter. Saleem et al. proposed a cut-off of 5.9 ± 2.7 for infections (vs. 4.1 ± 2.1 in non-infected grafts) [50], but, of course, these findings need to be further confirmed by larger studies and they need to be validated and standardized. At the moment, SUV and TBR analyses seem to have limited value in the assessment of VGI.

In a recently published meta-analysis exploring the accuracy and the efficacy of [^18^F]FDG PET/CT, the authors analyzed five different methods of interpretation of a PET/CT scan [39]. The sensitivity and specificity of qualitative assessment, using a five-point visual scale, were 89% and 61%, respectively; for focal uptake, they were 93% and 78%, respectively; for SUVmax, they were 98% and 80%; 57% and 76% for T/B ratio, respectively, and 100% and 88% for dual timepoint imaging (DTPI). However, only one paper investigated the added value of DTPI with additional delayed acquisitions and calculation of percentages of SUVmax change between initial and delayed images [44]. Despite the limitations of SUVmax, from this meta-analysis, it emerges that focal uptake and SUVmax are the most reliable tools for the interpretation of a PET/CT scan. Nevertheless, larger prospective and retrospective studies are needed to support these findings.

## 5. Consensus Statements from Round Table of 3rd European Congress of Infection and Inflammation

During the 3rd European Congress of Infection and Inflammation organized in Rome in December 2019, several specialists evaluating patients with VGI gave lectures on this topic from different points of view. Final discussions and a round table were carried out by the representatives of each specialty (C.L., R.I., M.R., G.T., A.S., M.T., P.A.E., Y.T), who also contributed to the present review. Although not officially endorsed by the respective European Societies of NM (EANM), Radiology (ESR) and Vascular Surgery (ESVS), here we summarize several statements that emerged from the round table of the Congress and that reached an oral consensus among these different specialists, aiming to provide evidence-based answers to the most frequent clinical questions.

### 5.1. In Case of a Partial Resection Graft for a Fragile Patient Unfit for a Total VGI Explantation, the Exact Infection Graft Location Could Be Useful for the Surgical Strategy. Which Radiological Integration Is More Precise in This Diagnosis?

Once a WBC scintigraphy or a [^18^F]FDG PET/CT scan clearly demonstrates that infection is not extended to the entire graft and perigraft tissues, a partial explanation is taken into consideration by surgeons if invasiveness limitation is advisable. What the surgeons need to know is the graft patency, the exact location of perigraft tissue alterations, the extent of fluid collection, and whether it would be possible to perform ligations or surgical bypass. All of this information is easily and can currently be obtained with CTA. There is almost no role for MRI. Only in selected cases could it be necessary to resort to DSA and endovascular interventions like embolization or stent grafting before surgery.

### 5.2. Does CTA Still Play a Role in Diagnosing Vascular Graft Infections or Should It Be Considered Obsolete, Replaced by NM Imaging?

CTA is requested by clinicians as the first-choice imaging modality in cases of suspected VGI. The main role is still to be considered for excluding this eventuality. A lack of significant peri-prosthetic fluid collections or bubbles (and also other information) could be collected, such as structural graft alterations, angulations or thrombosis. In these cases, a “wait and see” strategy can be adopted. In the case of persistent symptoms and more founded suspicions, a second CTA is still indicated to ascertain the evolution of the previous findings. In the case of endografts, CTA is the best imaging modality for demonstrating ruptures, disconnections, displacements and endoleaks, which are conditions often associated with infections. Last but not least, fluid collection aspiration for biological tests and cultures is almost exclusively performed by interventional radiologists under CT guidance. Therefore, CTA still plays a critical role in the diagnosis of VGI as the first diagnostic imaging modality [7]. NM modalities are complementary and may be useful to map the extent of the infection. Therefore, for patients with a clinical suspicion of vascular graft/endograft infection and with non-convincing findings on CTA, the use of WBC scintigraphy or [^18^F]FDG PET/CT is recommended as an additional imaging modality to improve diagnostic accuracy.

### 5.3. Does Antibiotic Therapy Affect NM Exams Accuracy? Should Antibiotic Therapy Be Stopped before NM Exams? If Yes, How Long before?

The influence of ongoing antimicrobial treatment on the different NM modalities and, in particular, on radiolabeled WBC scintigraphy, is still a matter of debate. Although the use of antibiotics is frequently reported in several papers, the duration of treatment is not always mentioned and it is not linked to the outcome of WBC scintigraphy or [^18^F]FDG PET/CT. Moreover, data regarding VGI do not exist, therefore a definitive conclusion on this topic cannot be provided.

From other clinical contexts mainly focused on musculoskeletal infections, some authors suggested that antibiotic treatment does not affect the accuracy of radiolabeled WBC; however, it is well known that antimicrobial treatment may reduce the chemiotaxis of leukocytes, thus resulting in lower migration into infected sites. Therefore, when antimicrobial therapy is ongoing, it should be considered during the scan interpretation, whereas, if the patient is at the end of antibiotic treatment, it is a common practice in the NM department to delay the execution of WBC scintigraphy after 2 weeks of therapy withdrawal.

Although antimicrobial treatment is known to decrease the intensity of [^18^F]FDG uptake, in a recently published retrospective study aiming to assess whether [^18^F]FDG PET/CT performance for the diagnosis of infective processes could be affected by ongoing antibiotic therapy, no false negative cases were detected in the group of patients receiving the treatment, thus demonstrating that the accuracy of this modality is not influenced by antibiotic administration [52].

### 5.4. Is It Reasonable to Perform an [^18^F]FDG-PET/CT after a Positive WBC Scintigraphy?

The answer to this question may be extracted in the meta-analysis of Reinders Folmer, where pre and post-test probabilities of having VGI have been calculated for CTA, [^18^F]FDG PET, [^18^F]FDG PET/CT, WBC scintigraphy with only planar images and WBC scintigraphy with planar images + SPECT/CT [12]. Of these modalities, WBC scintigraphy combined with SPECT/CT acquisitions scored best in terms of positive post-test probability (96%), followed by WBC scintigraphy with only planar images (94%), [^18^F]FDG PET/CT (83%), CTA (80%) and standalone [^18^F]FDG PET (78%). It means that, after positive WBC scintigraphy + SPECT/CT, a patient suspected of having a VGI has a 96% probability of being infected. This is not surprising considering the high number of true positives detected by this modality, which is, of course, superior to the number of true positives identified by [^18^F]FDG PET/CT.

Therefore, we can assume that a positive WBC scintigraphy, especially if correctly acquired with SPECT/CT and interpreted by following EANM recommendations, is sufficient for the diagnosis and does not require an additional study with [^18^F]FDG PET/CT.

### 5.5. Which Imaging Modality Is Recommended Within the First 3 Months after Surgery in the Suspicion of Early Infection?

It is well known that inflammatory changes, such as non-infected hematoma or lymphocele, may occur and persist for months after surgery, especially in more invasive approaches, and may result in false positive cases at both [^18^F]FDG PET/CT scans and, more rarely, at WBC scintigraphy, thus impacting on their accuracy. Moreover, synthetic graft material (Dacron or Gore-Tex) induces a foreign-body reaction which may present [^18^F]FDG uptake, thus representing a frequent pitfall in the interpretation of a PET scan. Indeed, after surgery, some inflammatory cells, mainly macrophages and fibroblasts, may use glucose as a source of energy for completing the healing process; for this reason, [^18^F]FDG is taken up by the healing tissue.

In a large retrospective study performed by Keidar, as previously mentioned, the authors explored the [^18^F]FDG uptake in 107 non-infected grafts in relation to graft material and time elapsed from surgery for a follow-up of up to 16 years. In this wide interval of time, they found no substantial reduction in the metabolic activity shown by synthetic grafts, thus meaning that post-surgical flogosis could be detectable after many years following surgery [49]. However, the pattern could be helpful in differentiating a sterile inflammation from an infection, since diffuse homogeneous uptake is usually observed in the first condition, reflecting a low-grade inflammation. On the contrary, infections usually show focal uptake. These findings were also confirmed by Wassèlius and colleagues in 10 out of 12 grafts implanted in open surgery and one out of four patients who underwent an endovascular procedure (mean time interval from surgery: 5.8 years). Notably, only one of the 16 patients had an infection based on biochemical and clinical data [46].

In terms of the usefulness of radiolabeled WBC in the post-surgical period, it is well known that this modality has higher specificity and accuracy in differentiating a sterile flogosis from an infection, compared with [^18^F]FDG PET/CT. In 2006, Liberatore et al. found no false positive results in patients studied within 1 month after surgery, concluding that this modality is reliable to assess an infection in the earlier stages after endovascular surgery [53]. Of course, this conclusion could be affected by the type of population studied and, in particular, by the probability of having an infection or not, and larger studies are needed to confirm this finding.

In conclusion, we can state that the perfect timing to perform an NM examination mainly depends on the type of surgery (open vs. endovascular approach), clinical indication and pre-test probability of infection. After surgery, the presence of aseptic flogosis must always be taken into consideration, especially in the interpretation of an [^18^F]FDG PET/CT scan and, therefore, the evaluation of the pattern of uptake and CT abnormalities must be accurate in order to correctly interpret the exam.

Larger multicenter studies are needed in order to provide an evidence-based answer to this question.

## 6. Conclusions

Accurate diagnosis of VGI is challenging and requires a multimodality and multidisciplinary approach in order to ensure the best management of these patients. Several radiological and NM modalities are available, each one with its pros and cons. US is usually used for extracavitary graft infection, while CT/CTA is the first-choice imaging modality for intracavitary graft infection. However, CTA may present some limitations, particularly in low-grade infections. In cases of equivocal CTA, WBC scintigraphy or [^18^F]FDG PET/CT are recommended in order to improve diagnostic accuracy, but the use of appropriate interpretation criteria is mandatory.

The best diagnostic option would be to combine anatomical/radiological and functional imaging in order to obtain an earlier and more effective diagnosis, which should be mandatory for decision making and for defining the best treatment options.

Many efforts still need to be directed towards the definition of accurate algorithms that aim to make diagnostic approaches more uniform among different centers.

## Figures and Tables

**Table 1:** Summary of the most relevant reviews and meta-analysis on Nuclear Medicine (NM) modalities for imaging.

Paper	Imaging Modality	Sensitivity	Specificity
Annovazzi 2005 [31]	^99m^Tc-WBC ^111^In-WBC CT	97.7% 84.1% 75%	88.6% 79.4% 56.6%
Reinders Folmer 2018 [12]	[^18^F]FDG PET [^18^F]FDG PET/CT WBC (planar) WBC SPECT/CT CTA	94%	70%
		95%	80%
		90%	88%
		99%	82%
		67%	63%
Khaja 2013 [33]	^99m^Tc-WBC ^111^In-WBC [^18^F]FDG PET/CT	83.7% 83% 93.7%	97.5% 87% 75%
Kim 2019 [38]	[^18^F]FDG PET/CT	96%	74%
Rojoa 2019 [39]	[^18^F]FDG PET/CT:		
	1. graded uptake 2. focal uptake 3. SUVmax 4. T/B ratio 5. DTPI	89% 93% 98% 57% 100%	61% 78% 80% 76% 88%

White blood cell (WBC); computed tomography (CT); ^18^F-fluorodeoxyglucose positron emission tomography/computed tomography ([^18^F]FDG PET/CT); single-photon emission computed tomography (SPECT); computed tomography–angiography (CTA).

## Supplementary Materials

The following are available online at https://www.mdpi.com/2077-0383/9/5/1510/s1, Video S1: surgery.
